# Detection Rate of Porcine Circoviruses in Different Ages and Production Herds of Intensive Pig Farms in China

**DOI:** 10.3390/ani15101376

**Published:** 2025-05-09

**Authors:** Mingyu Fan, Zhiqiang Hu, Lujie Bian, Yunzhou Wang, Xiaoyang Zhang, Xiaowen Li, Xinglong Wang

**Affiliations:** 1 College of Veterinary Medicine, Northwest A&F University, Yangling 712100, China; keepitup9968@126.com; 2 Shandong Swine-Health-Station Agriculture and Animal Husbandry Technology Co., Ltd., Dezhou 253000, China; 3 Key Laboratory of Animal Epidemic Disease Detection and Prevention in Panxi District, College of Animal Science, Xichang University, Xichang 615013, China; zhiqianghu0624@163.com; 4 Shandong Engineering Research Center of Pig and Poultry Health Breeding and Important Infectious Disease Purification, Shandong New Hope Liuhe Group Co., Ltd., Qingdao 266100, China; lujiebian@163.com; 5 Department of Veterinary Medicine, Shandong Vocational Animal Science and Veterinary College, Weifang 261061, China; wyzwin01@163.com (Y.W.); yang050576@126.com (X.Z.); 6 College of Agriculture and Biology, Liaocheng University, Liaocheng 252000, China

**Keywords:** porcine circoviruses, qPCR, different age herds, detection rate, mixed infection, correlation

## Abstract

Porcine circoviruses (PCVs), encompassing porcine circovirus type 1 (PCV1), porcine circovirus type 2 (PCV2), porcine circovirus type 3 (PCV3), and porcine circovirus type 4 (PCV4), have been documented in China and represent a significant threat to the swine industry. Nevertheless, there is a paucity of data regarding the infection characteristics of PCVs across different age groups within intensive pig farming operations. In this investigation, a systematic cross-sectional methodology was employed to collect 415 testicular processing fluid samples and 1583 serum samples from 30 breeding farms and 27 fattening farms in China. All samples underwent analysis using real-time quantitative polymerase chain reaction (qPCR). Among the testicular fluid samples from suckling pigs, the detection rates for PCV1, PCV2, PCV3, and PCV4 were 56.9%, 31.1%, 75.4%, and 2.2%, respectively. The lowest mean cycle threshold (Ct) values for PCV1 and PCV3 were observed in testicular fluid as opposed to serum samples. At the individual level, the detection rate of PCV1 was significantly higher in fattening pigs (28.7%) and sows (28.7%) compared to nursery pigs (8.5%). The detection rate of PCV2 was highest in fattening pigs (43.1%) and lowest in sows (19.2%). The infection profile of PCV3 contrasted markedly with that of PCV2, exhibiting the lowest prevalence in fattening pigs (8.1%) and the highest in sows (46.1%). PCV4 was infrequently detected across all age groups, with prevalence rates ranging from 0% to 1.7%. Furthermore, the incidence of mixed infections involving the four PCV types was observed to be 12.7% in nursery pigs, 16.8% in fattening pigs, and 22.4% in sows. Notably, no strong correlation was identified between any two co-detected PCV types across all pig age categories. The findings of this study contribute valuable insights into the infection dynamics of PCVs across different pig age groups. Additionally, this research offers critical reference information for devising strategies to prevent PCV infections in intensive pig farming operations in China.

## Simple Summary

Porcine circoviruses (PCV1, PCV2, PCV3, and PCV4) are prevalent in China, with PCV2 and PCV3 being most frequently associated with clinical disease and contributing to substantial economic losses in pig farming. This study aimed to examine the infection characteristics of PCVs across different age groups in intensive pig farming operations in China. In samples of suckling pig testicular fluid, PCV3 exhibited the highest detection rate at 75.4%, followed by PCV1 at 56.9%, PCV2 at 31.1%, and PCV4 at 2.2%. At the individual level, PCV1 detection was lowest in nursery pigs. The detection rate of PCV2 was highest in fattening pigs and lowest in sows. Conversely, PCV3 infection was least prevalent in fattening pigs (8.1%) and most prevalent in sows (46.1%). PCV4 was infrequently detected across all age categories. Furthermore, the incidence of mixed infections involving the four PCV types was 12.7% in nursery pigs, 16.8% in fattening pigs, and 22.4% in sows. However, no strong correlation was identified between any two co-detected PCV types across all age groups. This study provides critical reference data for the formulation of strategies aimed at preventing PCV infections in pig farms.

## 1. Introduction

Circovirus is a small, nonenveloped virus characterized by a circular icosahedral single-stranded DNA genome and widely distributed in mammals, fish, avian species, and even insects [1,2]. In pigs, four distinct porcine circoviruses (PCVs) have been identified and are named porcine circovirus type 1 (PCV1), porcine circovirus type 2 (PCV2), porcine circovirus type 3 (PCV3), and porcine circovirus type 4 (PCV4) [3,4,5,6]. PCV1 was originally detected as a noncytopathic contaminant of the PK-15 cell line [3], and it is generally accepted that PCV1 is nonpathogenic to pigs [7]. Nevertheless, PCV1 is capable of efficient replication and can induce pathological changes in the lungs of porcine fetuses [8]. PCV2 is widespread and is the causative agent of porcine circovirus-associated disease (PCVAD) in pigs, resulting in substantial economic losses to the global swine industry [9]. In addition, PCV3 was first identified in 2015 [5] and has been associated with several clinical–pathological conditions, such as porcine dermatitis and nephropathy syndrome (PDNS) [10], porcine respiratory disease complex (PRDC) [11], congenital tremors [12] or reproductive disorders [13,14], and multisystem inflammation [15]. Retrospective studies have indicated that PCV3 has likely been circulating for an extended period rather than having recently emerged and progressively spread across various countries [16]. In 2019, PCV4 was found in several pigs with severe clinical disease in China [6]. Currently, PCV4 has been reported in several countries, including China, Republic of Korea, Thailand, Spain, and the United States [17,18,19,20,21]. The pathogenicity and effect of PCV4 on pigs remain ambiguous currently. However, reported clinical manifestations associated with the detection of PCV4 include porcine dermatitis and nephropathy syndrome (PDNS), postweaning multisystemic wasting syndrome (PMWS), neurological signs, diarrhea, enteritis, encephalitis, respiratory disease, and reproductive disorders, as well as subclinical infection [21]. Notably, the mixed infection with two or more PCV pathogens is often found in clinical cases, with potential implications for disease severity and pathogenesis [18,21,22,23,24,25,26].

All four types of PCVs have been identified in pigs in China [6,23,27,28,29,30]. Previous studies have reported varying positivity rates across different regions: PCV1 ranges from 4.17% to 14.80% [27,31,32], PCV2 from 8.31% to 72.90% [23,26,28,33,34,35], PCV3 from 5.10% to 80.00% [26,31,32,35,36], and PCV4 from 3.33% to 45.39% [6,22,32,37,38]. Additionally, PCVs have been detected in a wide range of age herds, including sows and suckling, nursery, and finishing pigs [15,21,34,39]. The proportion of intensive pig farms in China is increasing year by year, but comprehensive information on the infection characteristics of the four PCVs in pig herds of different ages in intensive pig farms is lacking. In this study, samples were collected from intensive pig farms to investigate the infection characteristics of PCVs in herds of different ages. The findings may offer valuable insights for developing strategies to prevent and control PCVs in intensive pig farming operations in China.

## 2. Materials and Methods

### 2.1. Farms and Animals

A cross-sectional study was carried out from January to March in 2024 in 14 provinces in China, with samples collected from 30 breeding (farrow–wean) and 27 fattening (wean–finish) farms, and consent was obtained from the owners of all sampled farms. The breeding farms varied in sow herd size (from 1000 to 3000), and all pigs were vaccinated against PCV2 when they were gilts. The fattening farms had a production scale of over 4000 pigs, and pigs were vaccinated with the PCV2 vaccine at 14–21 days of age. Detailed information on the examined farms is shown in the Supplementary Materials (Tables S1 and S2).

### 2.2. Sample Collection

In each breeding farm, the collection of testicular processing fluid samples was undertaken during piglet castration at 3–5 days of age. Testicle samples from 20 litters were pooled into a single plastic bag, and the resulting fluid was transferred into a plastic tube. Meanwhile, 30 blood samples were randomly drawn from sows, incubated at room temperature for 30 min, and centrifuged at 1000× *g* for 2 min to obtain serum samples. In each fattening farm, 15 serum samples were randomly collected from nursery pigs (7–10 weeks old) and fattening pigs (14–20 weeks old), respectively. With these numbers, 30 serum samples of sows collected from breeding farms and 30 of nursery and fattening pigs from fattening farms, any agent could be detected if present in ≥10% of the animals in the farm units (95% confidence). In this study, 415 testicular processing fluid samples and 1583 serum samples were collected. All collected samples were stored at −20 °C.

### 2.3. qPCR Detection of PCVs

Serum and other liquid samples were oscillated and centrifuged at 5000× *g* for 1 min. Total DNA was extracted from 200 µL of each sample using a Virus DNA Extraction Kit II (Geneaid, New Taipei City, Taiwan) in accordance with the manufacturer’s instructions. A quadruplex real-time qPCR was used to detect the presence of the PCV1, PCV2, PCV3, and PCV4 DNA in each examined sample as previously described [32]. Briefly, the ORF1 of PCV1 was detected using forward primer 5′-AACCCCATAAGAGGTGGGTGTT-3′ and reverse primer 5′-TTCTACCCTCTTCCAAACCTTCCT-3′ with the probe 5′-TAMRA-TCCGAGGAGGAGAAAAACAAAATACGGGA-BHQ2-3′. The ORF1-ORF2 of PCV2 was detected using forward primer 5′-CTGAGTCTTTTTTATCACTTCGTAATGGT-3′ and reverse primer 5′-ACTGCGTTCGAAAACAGTATATACGA-3′ with the probe 5′-ROX-TTAAGTGGGGGGTCTTTAAGATTAAATTCTCTGAATTGT-BHQ2-3′. The ORF2 of PCV3 was detected using forward primer 5′-CATAAATGCTCCAAAGCAGTGCT-3′ and reverse primer 5′-TCACCCAGGACAAAGCCTCTT-3′ with the probe 5′-HEX-ATATGTGTTGAGCCATGGGGTGGGTCT-BHQ1-3′. The ORF1-ORF2 of PCV4 was detected using forward primer 5′-ATTATTAAACAGACTTTATTTGTGTCATCACTT-3′ and reverse primer 5′-ACAGGGATAATGCGTAGTGATCACT-3′ with the probe 5′-FAM-ATACTACACTTGATCTTAGCCAAAAGGCTCGTTGA-BHQ1-3′. The amplification condition was 95 °C for 30 s followed by 40 cycles of 95 °C for 5 s and 60 °C for 1 min. Fluorescence signal was determined at the end of each cycle of the 60 °C extension step. Samples with Ct values of <40 were considered positive.

### 2.4. Statistical Analysis

Statistical analyses were performed using SPSS Statistics version 22 (IBM Corp., Armonk, New York, NY, USA). The chi-squared test (with Bonferroni adjustments) was used to test for differences in the proportions positive for PCVs. The detection rates of PCV in different age categories were represented as absolute and relative frequencies (%) with a 95% confidence interval. The PCV DNA mean Ct values of each pig herd were analyzed using a one-way ANOVA, with a *p*-value < 0.05 being considered significant. For each type of PCV, its presence or absence in the sample was coded as 1/0. The strength of the correlation for the presence of different PCVs at sample level was assessed using the phi correlation coefficient. A *p* value < 0.05 was considered statistically significant.

## 3. Results

### 3.1. Distribution of the Examined Farms of PCVs Found

In breeding farms, PCV1 and PCV3 were detected in all examined farms (30 out of 30), PCV2 was detected in 29 out of the 30 farms, and PCV4 in 4 out of the 30 farms. In contrast, in the fattening farms, PCV1, PCV2, PCV3, and PCV4 were present in 21 out of the 27, 23 out of the 27, 15 out of the 27, and 2 out of the 27 farms, respectively. Across 57 farms, 10 distinct virus combination patterns were observed (Table 1); the percentage of positive samples in each farm is shown in Tables S1 and S2. Of the 30 breeding farms, all types of PCV were detected in 4 farms, while a combination of PCV1, PCV2, and PCV3 was detected in 25 farms. Only one breeding farm detected PCV1 and PCV3. Of the 27 fattening farms, 10 farms detected PCV1, PCV2, and PCV3, and 8 farms detected PCV1 and PCV2. only one fattening farm detected all types of PCVs, while one fattening farm did not detect any PCVs.

### 3.2. PCV Detection Rates in Different Age Categories of Pigs

**Figure 1:**
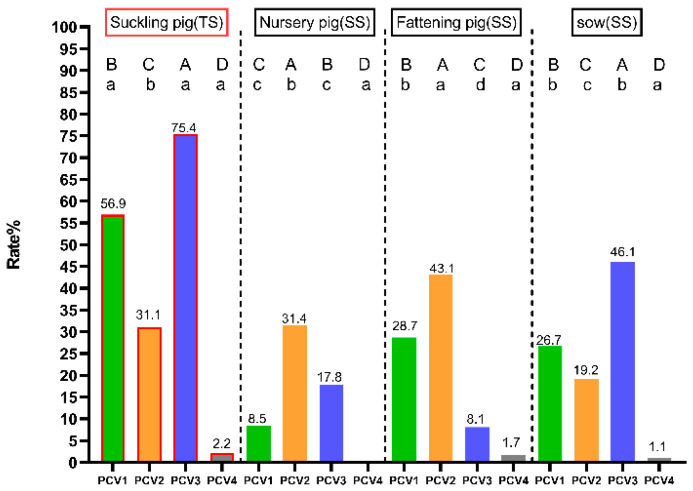
The detection rates of PCVs in different pig age categories. Superscript letters A, B, C, and D indicate significant differences in the detection rates of the same pig age categories for the four types of PCV. Superscript letters a, b, c, and d indicate significant differences in the detection rate of a type of PCV in different age categories of pigs. Different letters indicate significant statistical differences (*p* < 0.05), and the same letter indicates no significant statistical differences (*p* > 0.05). The red frame indicates that the sample is a pooled sample. Abbreviations: TS, testicular processing fluid sample; SS, serum sample.

Figure 1 illustrates the detection rates of PCVs across different age categories of pigs. In suckling piglets, the detection rate of PCV3 in testicular processing fluid samples was the highest at 75.4% (95% CI: 71.3–79.6%) (*p* < 0.05), followed by PCV1 (56.9%, 95% CI: 52.1–61.7%) and PCV2 (31.1%, 95% CI: 26.6–35.6%). On the other hand, it was observed that the detection rates of PCV3 and PCV1 in testicular processing fluid samples were significantly higher than those in serum samples (*p* < 0.05). In nursery pigs (31.4%, 95% CI: 25.4–37.3%) and fattening pigs (43.1%, 95% CI: 38.4–47.9%), the detection rate of PCV2 was highest compared to other PCV types (*p* < 0.05). In sows, PCV3 had the highest detection rate (46.1%, 95% CI: 42.8–49.3%). For PCV1, the detection rate was significantly higher in fattening pigs (28.7%, 95% CI: 24.3–33.0%) and sows (26.7%, 95% CI: 23.8–29.6%) than in nursery pigs (8.5%, 95% CI: 4.9–12.1%) (*p* < 0.05). The detection rate of PCV2 was the highest in fattening pigs (43.1%, 95% CI: 38.4–47.9%) (*p* < 0.05), followed by nursery pigs (31.4%, 95% CI: 25.4–37.3%) and sows (19.2%, 95% CI: 16.7–21.8%). In contrast, the detection rate of PCV3 was the highest in sows (46.1%, 95% CI: 42.8–49.3%) (*p* < 0.05), followed by nursery pigs (17.8%, 95% CI: 12.9–22.7%) and fattening pigs (8.1%, 95% CI: 5.4–10.7%). However, the detection rate of PCV4 ranged from 0.0% to 2.2% (95% CI: 0.8–3.6%) in different age categories, which was the lowest detection rate of all PCVs (*p* < 0.05).

### 3.3. The Ct Value of PCVs in Different Age Categories of Pigs

**Figure 2:**
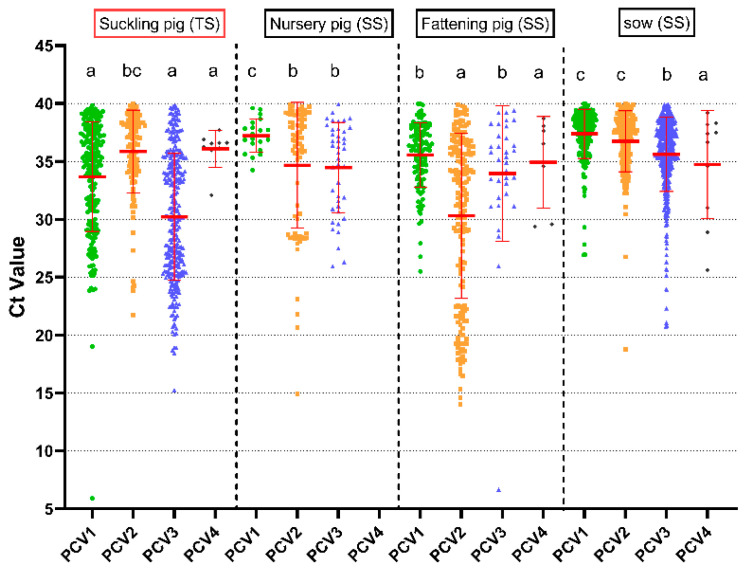
Ct values of PCV DNA-positive samples in different age categories. Superscript letters a, b, c, and d indicate significant differences in the mean Ct values of one PCV across different age pig herds. Different letters indicate significant statistical differences (*p* < 0.05), and the same letter indicates no significant statistical differences (*p* > 0.05). The red frame indicates that the sample is a pooled sample. Abbreviations: TS, testicular processing fluid sample; SS, serum sample.

The distribution of Ct values for PCV1, PCV2, PCV3, and PCV4 positive samples is shown in Figure 2, with low Ct values indicating higher amounts of viral DNA copies. In the testicular fluid sample from suckling piglets, the mean Ct value of PCV3-positive samples was 30.2 ± 5.5, which is significantly lower than that of the other PCV types (*p* < 0.05). In addition, lower Ct values of PCV1 (33.7 ± 4.7) and PCV3 (30.2 ± 5.5) were observed in the testicular fluid sample than in serum samples from other pig herds (*p* < 0.05). For PCV1, the mean Ct value of fattening pigs was 35.57 ± 2.8, while that of nursery pigs and sows was 37.22 ± 1.4 and 37.22 ± 2.1. For PCV2, the lowest Ct values of PCV2 were observed in fattening pigs (30.3 ± 7.1) compared to nursery pigs (34.7 ± 5.4) and sows (36.7 ± 2.6) (*p* < 0.05), and a similar trend was observed for PCV1. However, there was no significant difference in the Ct values of PCV3-positive serum samples in the different age categories (*p* > 0.05). In this study, the number of PCV4-positive samples was very low, and there were few samples with low Ct values.

### 3.4. The Distribution of PCV Mixed Infection Combinations

The distribution of PCV mixed infection combinations at the individual level is shown in Table 1. In nursery and fattening pigs, the highest single pathogen detection rate was PCV2 (18.6% and 27.5%, respectively), but in sows it was PCV3 (27.0%). In nursery pigs, the rate for the PCV2 + PCV3 combination was 8.9%, followed by PCV1 + PCV2 with 3.4%. In addition, the tri-pathogen combination “PCV1 + PCV2 + PCV3” was detected in only one pig (0.4%). In fattening pigs, the higher rates of “PCV1 + PCV2” (9.7%), “PCV2 + PCV3”, and “PCV1 + PCV3” were 2.8% and 1.2% respectively. The rate of PCV1 + PCV2 + PCV3 was 1.7% in fattening pigs. In sows, the rate of “PCV1 + PCV3” was higher (8.4%), followed by “PCV2 + PCV3” (6.2%) and “PCV1 + PCV2” (3.2%). Notably, the tri-pathogen combination with “PCV1 + PCV2 + PCV3” was found in 3.9% of the sows, and 0.3% of the sows (three sows) had a rare infection with four PCV types. The detection rate of PCV4 is low, so the probability of mixed infection with PCV1, PCV2, and PCV3 is very low. Overall, the rate of mixed infection between PCVs in nursery pigs, fattening pigs, and sows was 12.7%, 16.8%, and 22.4%, respectively.

### 3.5. The Presence Correlation Between Different PCV Types in Age Categories of Pigs at the Individual Level

The proportion of any two pathogens co-detected among the four PCVs and the phi correlation coefficient were calculated to determine the presence associations between different PCV types at the individual level and are shown in Table 2. In nursery pigs, the co-detection rate of PCV2 and PCV3 was the highest (9.3%) but showed a weak correlation between them (r = 0.21; *p* < 0.05). The co-detection rates for PCV1 and PCV2 and for PCV1 and PCV3 were 3.8% and 0.4%, respectively, with no significant correlation observed (*p* > 0.05). Among fattening pigs, the co-detection rates were 11.8% for PCV1 and PCV2, 4.7% for PCV2 and PCV3, and 2.8% for PCV1 and PCV3, with no significant correlations detected (*p* > 0.05). PCV4 exhibited low co-infection rates with PCV1, PCV2, and PCV3, which were 0.5%, 1.4%, and 0.2%, respectively. The presence of PCV4 was very weakly correlated with PCV2 (r = 0.11, 0.08; *p* < 0.05). In sows, the highest co-infection rate was observed in PCV1 and PCV3 at 12.6%, followed by PCV2 and PCV3 at 10.5%, and PCV1 and PCV2 at 7.6%. PCV2 infection showed a very weak correlation with PCV1 and PCV3 (r = 0.14, 0.08; *p* < 0.05). PCV4 also had low co-infection rates with PCV1, PCV2 and PCV3, all at 0.5%. Overall, no strong correlation was found between the presence of PCV at the individual level in nursery pigs, fattening pigs, and sows.

## 4. Discussion

The findings from previous epidemiological surveys suggest that PCV2 and PCV3 are prevalent across various regions in China [23,26,28,29,31,33]. These findings align with the outcomes of the present study, which detected PCV2 and PCV3 in the majority of pig farms. Although PCV1 is frequently identified in numerous investigations, the incidence of PCV1 DNA-positive pigs is generally low [27,31,32]. Nonetheless, similar to PCV2 and PCV3, PCV1 was identified in most intensive farming operations in this study, with a notable prevalence observed in finishing pigs and sows. It is common for PCV1, PCV2, and PCV3 to coexist on farms. Research conducted in Henan Province has reported high positive rates for PCV4, ranging from 25.4% to 45.39% [30,37,38], whereas studies from other regions, including the current study, indicated lower positive rates [22,23,32]. This suggests that despite the generally low prevalence of PCV4 across most regions of China, there remains a potential for localized expansion of the infection. Notably, this study identified a high positive rate in one breeding farm (Farm B24) and one fattening farm (Farm F20) (Tables S1 and S2), implying that PCV4, akin to PCV2, has the potential to cause widespread infection within affected farms.

PCVs have been detected in cases of aborted or stillborn piglets as well as newborn pigs [15,17,40,41,42,43], indicating the possible occurrence of vertical transmission and early infection of piglets with PCVs. Testicular fluid samples have been shown to be effective in the detection of various porcine pathogens in breeding farms, including porcine reproductive and respiratory syndrome virus [44], atypical porcine pestivirus [45], PCV2 [34], and PCV3 [39]. The positive rates for PCV1, PCV2, and PCV3 in testicular fluid samples in this study were high, indicating that the early infection with these viruses is a common occurrence in piglets. In addition, the presence of virus in the testicular fluid suggests active virus circulation between sows and newborn piglets and transplacental infection [39]. Notably, piglets carrying these pathogens serve as significant sources of infection for downstream fattening farms. Although PCV1 is generally considered nonpathogenic [40,46], one study found that PCV1 can replicate efficiently and produce pathology in the lungs of porcine fetuses, with this pathology being strongly correlated with elevated PCV1 titers in the lungs [8]. Notably, the high detection rate and viral load of PCV1 in testicular fluid samples from suckling pigs was observed in this study. Therefore, the potential disease impact on fetuses and suckling pigs requires further investigation of PCV1 in intensive pig farms in China.

In the current investigation, the detection rates of PCV2 in the testicular fluid and serum of sows were found to be 31% and 19.2% (Figure 1), respectively, surpassing previously reported figures [33,34]. The absence of PCV2 vaccination in the sows on the studied farms may have contributed to the elevated detection rates of PCV2 in both sows and their newborn piglets [47,48]. Incorporating PCV2 vaccination into the immunization protocols of breeding farms could potentially mitigate viremia and tissue load of PCV2 in offspring, thereby preventing PCV2 systemic disease (PCV2-SD) [47,49,50,51]. Conversely, the detection rate of PCV2 in testicular fluid samples was lower compared to that of PCV1 and PCV3. Despite the lack of PCV2 vaccination for the sows in this study, they had received two doses of the PCV2 vaccine during their gilt stage, which may have contributed to a reduction in PCV2 infection in their progeny [48]. Currently, no commercial vaccines are currently available for the control of PCV1 and PCV3 infections, apart from PCV2. Numerous studies have demonstrated the advantageous effects of PCV2 vaccination in field conditions, including reductions in PCV2 viremia and viral shedding, as well as enhancements in performance parameters [52,53,54]. In this study, elevated detection rates and viral loads of PCV2 were observed in the serum of fattening pigs despite vaccination against PCV2 at 14–21 days of age. It is important to highlight that elevated serum PCV2 loads correlate with an increased risk of PCVAD [9,55]. Consistent with these findings, several field studies reported that vaccination at 3–4 weeks of age offers enhanced protection against early infection, but a higher percentage of PCV2-positive pigs still occurred during the fattening phase [56,57,58]. Given the substantial infection pressure during the fattening stage, a single dose of the PCV2 vaccine appears insufficient to confer protection throughout this period. Conversely, numerous studies indicate that administering a double vaccination against PCV2 in piglets may enhance viral protection [59,60,61]. Furthermore, increasing the frequency of PCV2 vaccinations in both sows and piglets may effectively reduce viremia and viral shedding associated with PCV2 clinical disease [57,62]. Overall, the high rates of PCV2 positivity and viral load observed in this study underscore the need to reassess the current PCV2 vaccination protocol.

PCV3 infection has been associated with various clinicopathological conditions, with reproductive failures in sows being the most frequently observed [13,63,64,65]. Additionally, PCV3 has been implicated in respiratory disorders, diarrhea, early mortality, and myocarditis in neonatal piglets [5,64,66,67,68]. Notably, the viral load of PCV3 transmitted vertically was a critical determinant of clinical outcomes, as piglets with high viral loads experienced clinical manifestations such as loss and death that could not be mitigated by passive immunity [68]. In this study, a high detection rate and viral load of PCV3 were identified in the testicular processing fluid samples of suckling pigs. Furthermore, the detection rate was highest in sows compared to nursery and fattening pigs, indicating a high prevalence across most breeding farms. Consequently, the development of a PCV3 vaccine aimed at immunizing sows to reduce infections and vertical transmission is of urgent importance. Interestingly, PCV3 and PCV2 exhibited completely opposite infection trends across all age groups within the herds. The detection rate of PCV2 was highest in the fattening phase, which may be explained by the influence of the vaccine on PCV2 considering that the vaccination of piglets significantly delayed the development of PCV2 viremia in the nursery phase [58]. However, under natural exposure, the high frequency of PCV2 positivity observed in weaners and then decreasing in older animals is similar to that of PCV3 in this study [69,70]. In sows, the low detection rate of PCV2 may have decreased due to the vaccination program against PCV2 infection during the gilt phase. On the other hand, compared with PCV2, PCV3 is more active in infecting the fetuses [71], which may further increase the activity of PCV3 in sows. In this context, a notable aspect of our study findings is the differential susceptibility to infection exhibited by PCV2 and PCV3 across various age groups within herds. This observation implies that future strategies and targets for controlling PCV3 may need to be distinct from those for PCV2.

Mixed infections involving PCVs have been documented in both clinically healthy and diseased pigs under field conditions [15,17,31], and our study identified various combinations of mixed infections in individual animals. The incidence of single infections with PCV2 was highest among nursery (18.6%) and fattening (27.5%) pigs, whereas PCV3 single infections were most prevalent in sows (27.0%) (Table 1). However, the most frequently detected co-infection combinations varied with herd age: PCV2 + PCV3 (8.9%) in nursery pigs, PCV1 + PCV2 (9.7%) in fattening pigs, and PCV1 + PCV3 (8.4%) in sow herds. Previous studies reported triple detection rates for PCVs ranging from 0.22% to 2.87% [31]. In the present study, the rates of triple infection with PCV1, PCV2, and PCV3 in nursery pigs, fattening pigs, and sows were found to be 0.4%, 1.7%, and 3.9%, respectively, with other combinations of triple infections being rare. Quadruple infections involving all PCVs were observed in only three sows. The overall rates of mixed infection with different PCVs were 12.7% in nursery pigs, 16.8% in fattening pigs, and 22.4% in sows. Consequently, mixed infections among various PCV types are not uncommon, particularly in sow herds.

PCV2 is known to target immune system cells directly and is considered immunosuppressive [72,73], while PCV3 is capable of replicating in nearly all tissues, with a preference for immune cells, where it induces targeted cell damage, such as apoptosis and immune suppression [74]. Similarly, PCV4 can replicate in lymphocytes and macrophages, both of which are associated with the immune system [21]. Thus, it is pertinent to investigate whether infection with one type of PCV increases the susceptibility of infection with another type. In this study, the co-detection rates of any two distinct types of PCVs co-detected ranged from 0.2% to 12.6% across all age categories, with no strong correlation identified between the presence of PCV1, PCV2, PCV3, and PCV4 at the individual level. On the other hand, PCV pathogens that exhibited high detection rates in herds of varying ages were more frequently co-detected. This suggests that each PCV type exhibits distinct and independent circulation patterns within pig herds, and the occurrence of co-infections may merely reflect the pervasive presence of PCV1, PCV2, and PCV3 across different herds rather than indicating any potential synergistic interactions. In addition, the homology among the four types of PCV is limited [75]. Therefore, it is recommended that future control measures for the four distinct types of PCV involve the development of targeted vaccines and the implementation of different vaccination and biosafety protocols.

A limitation of this study is that the pig farms examined employed a similar low-frequency PCV2 vaccination strategy, and this study did not include pig farms utilizing alternative PCV2 vaccination strategies, which may impact the comprehensiveness of the PCV2 detection rate results. However, since the introduction of African swine fever into China [76], basic vaccines, including PCV vaccine, have been administered with less frequency on many intensive farms to mitigate the risk of disease introduction or cross-infection through personnel contact with pigs [33]. Therefore, the findings related to PCV2 in this study remain representative and hold reference importance. It is important to note that this study was conducted from January to March and possesses a cross-sectional nature, leaving uncertainty as to whether these results would vary in other months. Nonetheless, significant deviations in our conclusions are not anticipated given that PCV has been demonstrated to persist and infect animals year-round [77], and modern swine farms typically maintain a well-regulated climate within barns throughout the year. Another limitation of our study is the lack of representation of all types of pig farms across all provinces in China. The objective of this research was not to conduct a comprehensive analysis of the prevalence of PCV2 across China. Instead, we collected samples from 57 intensive pig farms of varying sizes across 14 major pig farming provinces in China. The aim was to assess the infection status of PCVs in pig farms with different herd ages and production herds. The resulting data are intended to provide intensive pig farms with evidence-based strategies for optimizing vaccination schedules, improving biosecurity protocols, and implementing targeted intervention measures.

## 5. Conclusions

In conclusion, the findings indicate that the prevalence of PCV1, PCV2, and PCV3 is higher in sows and their offspring, underscoring the necessity of implementing preventative measures in breeding herds to mitigate infection. Notably, PCV2 and PCV3 exhibit distinct susceptibilities to infection across different age groups within herds. Although mixed infections involving various PCV types are relatively common, no strong correlation was observed between the co-existence of any two PCV2 types at the individual level. This study offers valuable insights into the infection dynamics of PCVs across different age groups in pigs and serves as an important reference for developing strategies to prevent PCV infections in intensive pig farming operations in China.

## Figures and Tables

**Table 1:** Percentage of combinations of different types of PCVs at farm and individual level.

PCV Types	Number and Percentage of PCV Combinations									
	Breeding Farm		Fattening Farm		Nursery Pig		Fattening Pig		Sow	
	Farm Number	Rate	Farm Number	Rate	Sample Number	Rate	Sample Number	Rate	Sample Number	Rate
Negative	0	0.00%	1	3.70%	131	55.50%	157	37.20%	316	34.20%
PCV1 only	0	0.00%	1	3.70%	11	4.70%	68	16.10%	98	10.60%
PCV2 only	0	0.00%	2	7.41%	44	18.60%	116	27.50%	50	5.40%
PCV3 only	0	0.00%	1	3.70%	20	8.50%	9	2.10%	250	27.00%
PCV4 only	0	0.00%	0	0.00%	0	0.00%	1	0.20%	3	0.30%
PCV1 + PCV2	0	0.00%	8	29.63%	8	3.40%	41	9.70%	30	3.20%
PCV1 + PCV3	1	3.33%	1	3.70%	0	0.00%	5	1.20%	78	8.40%
PCV1 + PCV4	0	0.00%	0	0.00%	0	0.00%	0	0.00%	1	0.10%
PCV2 + PCV3	0	0.00%	1	3.70%	21	8.90%	12	2.80%	57	6.20%
PCV2 + PCV4	0	0.00%	0	0.00%	0	0.00%	3	0.70%	0	0.00%
PCV3 + PCV4	0	0.00%	0	0.00%	0	0.00%	0	0.00%	1	0.10%
PCV1 + PCV2 + PCV3	25	83.33%	10	37.04%	1	0.40%	7	1.70%	36	3.90%
PCV1 + PCV2 + PCV4	0	0.00%	0	0.00%	0	0.00%	2	0.50%	1	0.10%
PCV1 + PCV3 + PCV4	0	0.00%	0	0.00%	0	0.00%	0	0.00%	0	0.00%
PCV2 + PCV3 + PCV4	0	0.00%	1	3.70%	0	0.00%	1	0.20%	1	0.10%
PCV1 + PCV2 + PCV3 + PCV4	4	13.33%	1	3.70%	0	0.00%	0	0.00%	3	0.30%
Total	30	100%	27	100%	236	100%	422	100%	925	100%

**Table 2:** The presence correlation between different PCV types in age categories of pigs at the individual level.

	PCV1 n (%), r	PCV2 n (%), r	PCV3 n (%), r	PCV4 n (%), r
Nursery Pig				
PCV1	20 (8.5%), 1			
PCV2	9 (3.8%), 0.09	74 (31.4%), 1		
PCV3	1 (0.4%), −0.1	22 (9.3%), 0.21 *	42 (17.8%), 1	
PCV4	/	/	/	/
Fattening Pig				
PCV1	123 (29.1%), 1			
PCV2	50 (11.8%), −0.32	182 (43.1%), 1		
PCV3	12 (2.8%), 0.04	20 (4.7%), 0.09	34 (8.1%), 1	
PCV4	2 (0.5%), −0.02	6 (1.4%), 0.11 *	1 (0.2%), 0.03	7 (1.7%), 1
Sow				
PCV1	247 (26.7%), 1			
PCV2	70 (7.6%), 0.14 *	178 (19.2%), 1		
PCV3	117 (12.6%), 0.02	97 (10.5), 0.08 *	426 (46.1%), 1	
PCV4	5 (0.5%), 0.06	5 (0.5%), 0.08 *	5 (0.5%), 0.08	10 (1.1%), 1

The table shows the results of the phi correlation matrices for the presence or absence of each PCV at the individual level. The strength of correlation for the presence of distinct PCVs was assessed by the phi correlation coefficient (r). *, *p* < 0.05, indicate the presence of distinct PCVs have correlation. |r| < 0.20 very weak correlation, 0.20 < |r| < 0.40 weak correlation, 0.40 < |r| < 0.70 moderate correlation, 0.70 < |r| < 0.90 strong correlation, and 0.90 < |r| < 1 very strong correlation. n, the number of samples co-detected by two different types of PCV.

## Data Availability

Data are provided within the article or Supplementary Information Files. The dataset generated in this study is available from the corresponding author on reasonable request.

## Supplementary Materials

The following supporting information can be downloaded at: https://www.mdpi.com/article/10.3390/ani15101376/s1, Table S1: Detailed information on the examined breeding farms and the percentage of positive samples in each farm; Table S2: Detailed information on the examined fattening farms and the percentage of positive samples in each farm.

## Abbreviations

The following abbreviations are used in this manuscript:

CI	Confidence interval
Ct	Cycle threshold
PCVs	Porcine circoviruses
PCV1	Porcine circovirus type 1
PCV2	Porcine circovirus type 2
PCV3	Porcine circovirus type 3
PCV4	Porcine circovirus type 4
PCVAD	porcine circovirus-associated disease
qPCR	Quantitative polymerase chain reaction
