# Supplementary material for: Detection Rate of Porcine Circoviruses in Different Ages and Production Herds of Intensive Pig Farms in China
Source: Animals (Basel). 2025 May 9;15(10):1376. doi: 10.3390/ani15101376 (PMC12108398; doi:10.3390/ani15101376)
Supplement: Supplementary file 1 [file animals-15-01376-s001.zip › Table S1.pdf]

Table S1. Detailed information on the examined breeding farms and the percentage of positive samples in each farm.

Breeding farm	Province	Sow (SS) Sample number	Sow (SS) Positivity rate (proportion of positive samples) PCV1	PCV2	PCV3	PCV4	Suckling pig (TS) Sample number	Suckling pig (TS) Positivity rate (proportion of positive samples) PCV1	PCV2	PCV3	PCV4
B1	Anhui	30	26.7% (8/30)	10.0% (3/30)	66.7% (20/30)	0.0% (0/30)	16	100.0% (16/16)	31.3% (5/16)	100.0% (16/16)	0.0% (0/16)
B2	Gansu	30	30.0% (9/30)	76.7% (23/30)	53.3% (16/30)	0.0% (0/30)	2	50.0% (1/2)	50.0% (1/2)	100.0% (2/2)	0.0% (0/2)
B3	Guangdong	30	16.7% (5/30)	10.0% (3/30)	43.3% (13/30)	0.0% (0/30)	16	18.8% (3/16)	6.3% (1/16)	100.0% (16/16)	0.0% (0/16)
B4	Guangxi	30	16.7% (5/30)	6.7% (2/30)	60.0% (18/30)	0.0% (0/30)	16	68.8% (11/16)	62.5% (10/16)	50.0% (8/16)	0.0% (0/16)
B5	Guangxi	30	20.0% (6/30)	23.3% (7/30)	23.3% (11/30)	0.0% (0/30)	22	100.0% (22/22)	63.6% (14/22)	100.0% (22/22)	0.0% (0/22)
B6	Guangxi	31	3.2% (1/31)	16.1% (5/31)	71.0% (22/31)	0.0% (0/31)	16	100.0% (16/16)	18.8% (3/16)	100.0% (16/16)	0.0% (0/16)
B7	Guangxi	29	10.3% (3/29)	10.3% (3/29)	17.2% (5/29)	0.0% (0/29)	16	93.8% (15/16)	18.8% (3/16)	56.3% (9/16)	0.0% (0/16)
B8	Guangxi	44	31.8% (14/44)	6.8% (3/44)	20.5% (9/44)	0.0% (0/44)	15	13.3% (2/15)	6.7% (1/15)	0.0% (0/15)	0.0% (0/15)
B9	Guizhou	30	40.0% (12/30)	6.7% (2/30)	36.7% (11/30)	0.0% (0/30)	22	72.7% (16/22)	50.0% (11/22)	45.5% (10/22)	0.0% (0/22)
B10	Guizhou	30	60% (18/30)	0.0% (0/30)	70% (21/30)	0.0% (0/30)	16	18.8% (3/16)	68.8% (11/16)	75.0% (12/16)	0.0% (0/16)
B11	Guizhou	30	30.0% (9/30)	10.0% (3/30)	30.0% (9/30)	0.0% (0/30)	16	100.0% (16/16)	6.3% (1/16)	100.0% (16/16)	0.0% (0/16)
B12	Hainan	30	3.2% (1/30)	6.7% (2/30)	53.3% (16/30)	0.0% (0/30)	16	18.8% (3/16)	75.0% (12/16)	100.0% (16/16)	0.0% (0/16)

B13	Hebei	30	40.0% (12/30)	76.7% (23/30)	16.7% (5/30)	0.0% (0/30)	16	18.8% (3/16)	37.5% (6/16)	43.8% (7/16)	0.0% (0/16)
B14	Henan	30	46.7% (14/30)	76.7% (23/30)	46.7% (14/30)	0.0% (0/30)	16	93.8% (15/16)	6.3% (1/16)	18.8% (3/16)	0.0% (0/16)
B15	Hubei	50	60.0% (30/50)	36.0% (18/50)	44.0% (22/50)	8.0% (4/50)	/	/	/	/	/
B16	Hunan	32	18.8% (6/32)	0.0% (0/32)	90.6% (29/32)	0.0% (0/32)	4	0.0% (0/4)	0.0% (0/4)	100.0% (4/4)	0.0% (0/4)
B17	Jiangsu	30	23.3% (7/30)	6.7% (2/30)	10.0% (3/30)	0.0% (0/30)	16	100.0% (16/16)	6.3% (1/16)	31.3% (5/16)	0.0% (0/16)
B18	Shandong	30	10.0% (3/30)	23.3% (7/30)	40.0% (12/30)	0.0% (0/30)	16	18.8% (3/16)	31.3% (5/16)	87.5% (14/16)	0.0% (0/16)
B19	Shandong	30	13.3% (4/30)	23.3% (7/30)	50.0% (15/30)	0.0% (0/30)	16	31.3% (5/16)	31.3% (5/16)	62.5% (10/16)	0.0% 00/16)
B20	Shandong	30	36.7% (11/30)	10.0% (3/30)	80.0% (24/30)	0.0% (0/30)	8	87.5% (7/8)	62.5% (5/8)	100.0% (8/8)	0.0% (0/8)
B21	Shandong	30	66.7% (20/30)	0.0% (0/30)	0.0% (0/30)	0.0% (0/30)	1	100.0% (1/1)	100.0% (1/1)	100.0% (1/1)	0.0% (0/1)
B22	Shandong	30	3.3% (1/30)	16.7% (5/30)	100% (30/30)	0.0% (0/30)	16	100.0% (16/16)	12.5% (2/16)	100.0% (16/16)	0.0% (0/16)
B23	Shandong	30	6.7% (2/30)	20.0% (6/30)	56.7% (17/30)	0.0% (0/30)	16	81.3% (13/16)	6.3% 1/16)	62.5% 10/16)	0.0% 0/16)
B24	Shandong	32	12.5% (4/32)	3.1% (1/32)	37.5% (12/32)	12.5% (4/32)	8	0.0% (0/8)	0.0% (0/8)	0.0% (0/8)	100.0% (8/8)
B25	Sichuan	30	6.7% (2/30)	10.0% (3/30)	43.3% (13/30)	0.0% (0/30)	16	93.8% (15/16)	43.8% (7/16)	93.8% (15/16)	0.0% (0/16)
B26	Sichuan	15	13.3% (2/15)	0.0% (0/15)	53.3% (8/15)	0.0% (0/15)	16	0.0% (0/16)	6.3% (1/16)	100.0% (16/16)	0.0% (0/16)
B27	Sichuan	30	53.3% (16/30)	26.7% (8/30)	70.0% (21/30)	3.3% (1/30)	16	0.0% (0/16)	62.5% (10/16)	100.0% (16/16)	0.0% (0/16)

B28	Sichuan	30	20.0% (6/30)	6.7% (2/30)	36.7% (11/30)	3.3% (1/30)	16	0.0% (0/16)	43.8% (7/16)	100.0% (16/16)	6.3% (1/16)
B29	Sichuan	32	37.5% (12/32)	12.5% (4/32)	12.5% (3/32)	0.0% (0/32)	13	15.4% (2/13)	0.0% (0/13)	100.0% (13/13)	0.0% (0/13)
B30	Zhejiang	30	13.3% (4/30)	33.3% (10/30)	53.3% (16/30)	0.0% (0/30)	16	100.0% (16/16)	25.0% (4/16)	100.0% (16/16)	0.0% (0/16)
Total	/	925	26.7% (247/925)	19.2% (178/925)	46.1% (426/925)	1.1% (10/925)	415	56.9% (236/415)	31.1% (129/415)	75.4% (313/415)	2.2% (9/415)

Abbreviations: TS, testicular processing fluid sample; SS, serum sample.
