# Supplementary material for: Detection Rate of Porcine Circoviruses in Different Ages and Production Herds of Intensive Pig Farms in China
Source: Animals (Basel). 2025 May 9;15(10):1376. doi: 10.3390/ani15101376 (PMC12108398; doi:10.3390/ani15101376)
Supplement: Supplementary file 1 [file animals-15-01376-s001.zip › Table S2.pdf]

Table S2. Detailed information on the examined fattening farms and the percentage of positive samples in each farm

Fattening Farm	Province	Nursery pig (SS)					Fattening pig (SS)				
		Sample number	Positivity rate (proportion of positive samples)				Sample number	Positivity rate (proportion of positive samples)			
			PCV1	PCV2	PCV3	PCV4		PCV1	PCV2	PCV3	PCV4
F1	Shandong	/	/	/	/	/	15	0.0% (0/15)	100.0% (15/15)	0.0% (0/15)	0.0% (0/15)
F2	Gansu	15	0.0% (0/15)	40.0% (6/15)	0.0% (0/15)	0.0% (0/15)	15	6.7% (1/15)	100.0% (15/15)	20.0% (3/15)	0.0% (0/15)
F3	Gansu	/	/	/	/	/	15	0.0% (0/15)	100.0% (15/15)	0.0% (0/15)	0.0% (0/15)
F4	Hainan	15	20.0% (3/15)	6.7% (1/15)	6.7% (1/15)	0.0% (0/15)	15	0.0% (0/15)	6.7% (1/15)	0.0% (0/15)	0.0% (0/15)
F5	Hubei	15	0.0% (0/15)	40.0% (6/15)	40.0% (6/15)	0.0% (0/15)	/	/	/	/	/
F6	Sichuan	15	0.0% (0/15)	0.0% (0/15)	13.3% (2/15)	0.0% (0/15)	15	0.0% (0/15)	6.7% (1/15)	26.7% (4/15)	6.7% (1/15)
F7	Guangxi	15	0.0% (0/15)	13.3% (2/15)	53.3% (8/15)	0.0% (0/15)	15	33.3% (5/15)	53.3% (8/15)	73.3% (11/15)	0.0% (0/15)
F8	Shandong	15	0.0% (0/15)	40.0% (6/15)	26.7% (4/15)	0.0% (0/15)	15	86.7% (13/15)	100.0% (15/15)	13.3% (2/15)	0.0% (0/15)
F9	Shandong	15	0.0% (0/15)	6.7% (1/15)	26.7% (4/15)	0.0% (0/15)	15	13.3% (2/15)	46.7% (7/15)	6.7% (1/15)	0.0% (0/15)
F10	Shandong	15	0.0% (0/15)	66.7% (10/15)	33.3% (5/15)	0.0% (0/15)	15	93.3% (14/15)	80.0% (12/15)	26.7% (4/15)	0.0% (0/15)
F11	Shandong	/	/	/	/	/	15	6.7% (1/15)	100.0% (15/15)	0.0% (0/15)	0.0% (0/15)

F12	Shandong	/	/	/	/	/	15	0.0%（0/15）	0.0%（0/15）	6.7%（1/15）	0.0%（0/15）
F13	Guangdong	/	/	/	/	/	15	6.7%（1/15）	20.0%（3/15）	6.7%（1/15）	0.0%（0/15）
F14	Guangxi	15	0.0%（0/15）	0.0%（0/15）	6.7%（1/15）	0.0%（0/15）	32	56.3%（18/32）	0.0%（0/32）	9.4%（3/32）	0.0%（0/32）
F15	Guizhou	/	/	/	/	/	15	26.7%（4/15）	93.3%（14/15）	0.0%（0/15）	0.0%（0/15）
F16	Guizhou	15	0.0%（0/15）	0.0%（0/15）	0.0%（0/15）	0.0%（0/15）	15	26.7%（4/15）	0.0%（0/15）	0.0%（0/15）	0.0%（0/15）
F17	Guizhou	16	0.0%（0/16）	6.3%（1/16）	0.0%（0/16）	0.0%（0/16）	15	93.3%（14/15）	0.0%（0/15）	0.0%（0/15）	0.0%（0/15）
F18	Henan	/	/	/	/	/	15	46.7%（7/15）	100.0%（15/15）	0.0%（0/15）	0.0%（0/15）
F19	Shandong	15	20.0%（3/15）	100.0%（15/15）	73.3%（11/15）	0.0%（0/15）	15	60.0%（9/15）	0.0%（0/15）	0.0%（0/15）	0.0%（0/15）
F20	Shandong	15	0.0%（0/15）	93.3.0%（14/15）	20.0%（3/15）	0.0%（0/15）	15	26.7%（4/15）	100.0%（15/15）	20.0%（3/15）	40.0%（6/15）
F21	Sichaun	15	66.7%（10/15）	20.0%（3/15）	0.0%（0/15）	0.0%（0/15）	15	6.7%（1/15）	0.0%（0/15）	0.0%（0/15）	0.0%（0/15）
F22	Hubei	/	/	/	/	/	15	6.7%（1/15）	26.7%（4/15）	0.0%（0/15）	0.0%（0/15）
F23	Hubei	/	/	/	/	/	15	66.7%（10/15）	6.7%（1/15）	0.0%（0/15）	0.0%（0/15）
F24	Hubei	/	/	/	/	/	15	0.0%（0/15）	0.0%（0/15）	0.0%（0/15）	0.0%（0/15）
F25	Guangdong	/	/	/	/	/	30	10.0%（3/30）	33.3%（10/30）	3.3%（1/30）	0.0%（0/30）

F26	Zhejiang	15	0.0%<br>（0/15）	0.0%<br>（0/15）	13.3%<br>（2/15）	0.0%<br>（0/15）	15	26.7%<br>（4/15）	13.3%<br>（2/15）	0.0%<br>（0/15）	0.0%<br>（0/15）
F27	Jiangsu	10	40.0%<br>（4/10）	70.0%<br>（7/10）	0.0%<br>（0/10）	0.0%<br>（0/10）	15	33.3%<br>（5/15）	93.3%<br>（14/15）	0.0%<br>（0/15）	0.0%<br>（0/15）
Total		236	8.5%<br>（20/236）	31.4%<br>（74/236）	17.8%<br>（42/236）	0.0%<br>（0/236）	422	28.7%<br>（121/422）	43.1%<br>（182/422）	8.1%<br>（34/422）	1.7%<br>（7/422）

Abbreviations: TS, testicular processing fluid sample; SS, serum sample.
